# In Vitro Activity of Water Extracts of Olive Oil against Planktonic Cells and Biofilm Formation of *Arcobacter*-like Species

**DOI:** 10.3390/molecules27144509

**Published:** 2022-07-14

**Authors:** Karolína Švarcová, Leona Hofmeisterová, Blanka Švecová, David Šilha

**Affiliations:** 1 Department of Biological and Biochemical Sciences, Faculty of Chemical Technology, University of Pardubice, Studentská 573, 532 10 Pardubice, Czech Republic; karolina.svarcova@student.upce.cz (K.Š.); leona.hofmeisterova@student.upce.cz (L.H.); 2 Department of Analytical Chemistry, Faculty of Chemical Technology, University of Pardubice, Studentská 573, 532 10 Pardubice, Czech Republic; blanka.svecova@upce.cz

**Keywords:** antimicrobial effect, *Arcobacter*-like, biofilm formation, HPLC, olive oil extract

## Abstract

Extra-virgin olive oils contain many bioactive substances that are phenolic compounds. The survival of *Arcobacter*-like strains in non-buffered (WEOO) and buffered (BEOO) extracts of olive oils were studied. Time kill curves of different strains were measured in the environment of olive oil extracts of different grades. The activity of the extracts was also monitored for biofilm formation using the Christensen method. In vitro results revealed that extra-virgin olive oil extracts exhibited the strongest antimicrobial effects, especially non-buffered extracts, which exhibited strain inhibition after only 5 min of exposure. The weakest inhibitory effects were observed for olive oil extracts. A decrease in biofilm formation was observed in the environment of higher WEOO concentrations, although at lower concentrations of extracts, increased biofilm formation occurred due to stress conditions. The dialdehydic forms of oleuropein derivatives, hydroxytyrosol, and tyrosol were the main compounds detected by HPLC-CoulArray. The results indicate that not all olive oils had a similar bactericidal effect, and that bioactivity primarily depended on the content of certain phenolic compounds.

## 1. Introduction

The first mention of arcobacters was recorded in 1977. However, they were originally mistaken for members of *Campylobacter* spp., later known as aerotolerant campylobacters [1]. According to recently identified differences in *Arcobacter* spp. from the genus *Campylobacter*, certain taxonomic changes have been proposed, including the designation of arcobacters as *Arcobacter*-like microorganisms [2,3]. Currently, *Arcobacter*-like microorganisms belong to a separate family of *Arcobacteraceae* [4]. It was also proposed to divide the original genus *Arcobacter* into different taxonomic units (*Aliarcobacter*, *Arcobacter*, *Halarcobacter*, *Malaciobacter*, *Poseidonibacter*, and *Pseudarcobacter*). At present, 29 species belong to *Arcobacter*-like microorganisms [2,5]. *Arcobacter*-like bacteria are Gram-negative, spiral, or slightly curved rods, and the size of the bacterial cells can vary (0.5–2 μm long and 0.4–0.6 μm wide). *Arcobacter*-like species are widespread, and they can be isolated from various samples [6]. Unlike the genus *Campylobacter*, arcobacters are not a natural part of the intestinal tract of poultry [7]. The association of arcobacters with human disease has been described for the species *Aliarcobacter butzleri*, *Aliarcobacter cryaerophilus*, *Aliarcobacter skirrowii*, and *Aliarcobacter thereius* species [8]. However, in other species, the pathogenic potential is being investigated [9]. These bacteria are able to form biofilms, which support their resistance to antibacterial substances and effects [10]. The increasing resistance of microorganisms is a serious and worldwide problem, which has stimulated research into new biologically active substances [11]. Herbal extracts and essential oils have been used in traditional medicines for thousands of years [12].

Olive oils can be categorized into different groups, i.e., virgin, refined, or blended oils. Virgin olive oil is one of the few edible vegetable oils that is consumed unrefined. That means it contains a significant amount of minor bioactive substances. Seed-free pressed oils are of the highest quality. Refined oil is pressed at high temperatures and pressures and then chemically treated. Blended oils include mixtures of olive oil and refined olive oil mixed in various proportions [13]. Furthermore, the olive oil obtained from pomace also belongs to the category of lower-quality oils. All olive oils contain almost the same amount of fatty acids, although the content of phenolic compounds varies significantly [14,15]. Virgin olive oils exhibited the highest antimicrobial activity, followed by olive oils and pomace oils [16,17]. However, water extract from olive oils also exhibited significant antimicrobial activity [18,19]. The many antimicrobial agents derived from olives include phenols or their substituted derivatives [20]. Phenolic compounds are capable of inhibiting the growth of many microorganisms, but the antimicrobial effect of different phenolic compounds varies [16]. One of the most important phenols found in olive oils is 3,4-dihydroxyphenylethanol (3,4-DHPEA). This phenolic compound is present in various oleosidic forms, e.g., it is associated with the dialdehyde form of elenolic acid or occurs as an isomer of oleuropein aglycone. All of these forms are also products of oleuropein degradation [21]. Other olive oil compounds with antimicrobial activity include hydroxytyrosol, tyrosol, oleuropein, and ligstroside. In particular, their aglycones and dialdehyde forms have strong bactericidal activity [17].

The aim of this study was to describe the antimicrobial activity of water extract from olive oils against *Arcobacter*-like strains. To our knowledge, there are no available studies focused on the antimicrobial activity of substances obtained from olive oils against *Arcobacter*-like strains. The antimicrobial activity of the extracts was evaluated by monitoring time kill curves. Additionally, the biofilm formation in the presence of the extracts was followed by a modified Christensen method. Chromatographic analysis of the prepared extracts was performed with the aim of their mutual evaluation and comparison, as well as to assess their biological effect.

## 2. Results

### 2.1. Chromatographic Analysis of the Extracts

The aqueous (WEOO) and buffered (BEOO) olive oil extracts of various quality grades were analyzed using the HPLC method. An HPLC system equipped with a CoulArray multichannel electrochemical detector was used as a selective and sensitive tool for the compounds with electrochemical behavior (e.g., bioactive polyphenolic compounds) that are present in olive oils.

**Figure 1:**
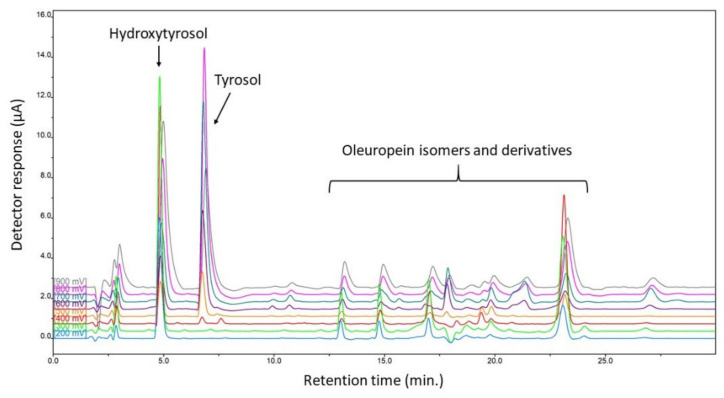
Chromatogram of WEOO of Ballester extra-virgin olive oil. Chromatographic conditions—Gemini C18 column (150 mm × 3 mm, 3 µm). Mobile phase A—5 mM ammonium acetate with formic acid (pH~3); mobile phase B—acetonitrile; flow rate 0.4 mL/min; gradient elution—0–30 min, 5–60% of mobile phase B; sample volume 10 µL; temperature 40 °C; detection at potentials 200–900 mV (step 100 mV).

The initial chromatographic conditions were chosen according to our previous results [22]. According to these, a Gemini C18 column (150 mm × 3 mm, 3 µm) was chosen, as well as other separation and detection conditions. Several gradient elution programs were tested to obtain good resolution of the target compounds in the extracts. The final chromatogram for one of the sample extracts is shown in Figure 1.

Hydroxytyrosol, tyrosol, and oleuropein isomers and derivatives were found to be the main components of the prepared oil extracts. The compounds were identified via comparison with the retention times and voltammetric behavior of standard compounds. The contents of hydroxytyrosol and tyrosol were determined using the calibration curve method, which was evaluated as the dependence of the peak area on the concentration. All oleuropein isomers and derivatives were expressed as the sum of peaks (as can be seen in Figure 1), and their content was related to standard oleuropein. The limit of detection (LOD) and the limit of quantification (LOQ) were established for the individual compounds. The LODs were determined using lower concentrations of standards for a S/N of 3:1 (S/N = 3). Baseline noise was evaluated by injecting the mobile phase (appropriate for each type of analysis) in five replicates. Similarly, the LOQs were calculated from a S/N of 10:1. The LOD and LOQ values for hydroxytyrosol, tyrosol, and oleuropein, together with calibration ranges, regression equations, and appropriate correlation coefficients R^2^, are summarized in Table 1.

Hydroxytyrosol, tyrosol, and total oleuropein derivatives contents were quantified in the water (WEOO) and buffered (BEOO) olive oil extracts. The results are presented in Table 2. The water extracts of the extra-virgin olive oils contained the highest amount of bioactive compounds. Specifically, in total, it was 71.4 mg/L in Spanish extra-virgin olive oil and 48.3 mg/L in Greek extra-virgin olive oil. The refined olive oil had a very low amount of monitored bioactive compounds due to losses during the refining process. Finally, in blended olive and sunflower oil (Borges), as well as olive pomace oil (Ondoliva), all the monitored bioactive compounds were below the LODs.

### 2.2. Total Polyphenol Content of the Extracts

Total polyphenol content (TPC) was measured using the spectrophotometric method with Folin–Ciocalteu reagent, and the results were expressed as gallic acid equivalent GAE (mg/L) [23], as presented in Table 3. The total polyphenol content exhibited the same trend among the samples as the results obtained via HPLC. Extra-virgin olive oil extracts exhibited the highest TPC (43.50–93.23 mg/L), followed by a sample of a blended sunflower and olive oil, olive pomace oil, and refined olive oil. In general, the TPC was always higher for samples extracted in PBS buffer (BEOO).

### 2.3. Antimicrobial Activity of Oil Extracts—Time Kill Curves of BEOO

**Figure 2:**
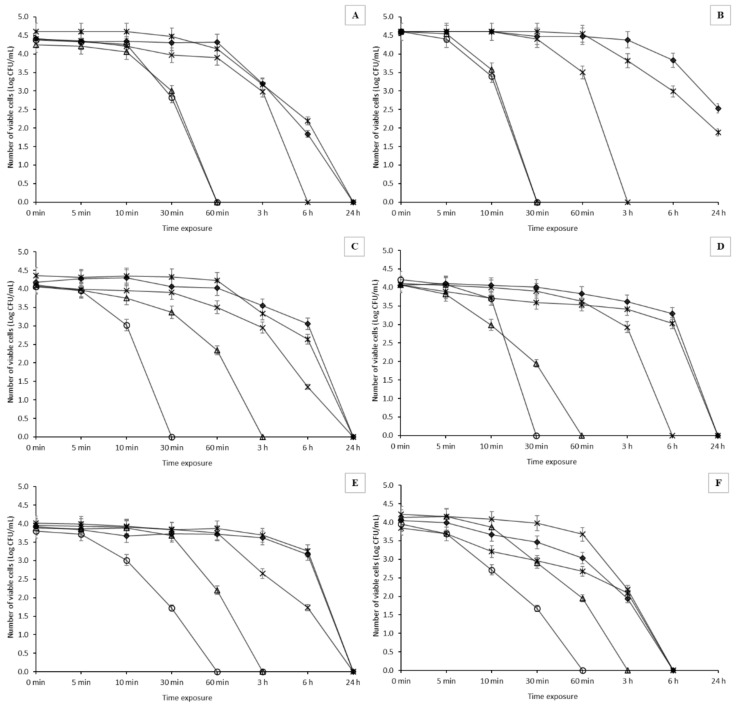
Survival of *Arcobacter*-like strains in the presence of BEOO at a sample concentration of 90%. Spanish extra-virgin olive oil (circle); Greek extra-virgin olive oil (triangle); blended sunflower and extra-virgin olive oil (times); pure olive oil (diamond); olive pomace oil (asterisk). (**A**)—*Aliarcobacter butzleri* CCUG 30484; (**B**)—*Aliarcobacter butzleri* UPa 2013/30; (**C**)—*Aliarcobacter cryaerophilus* CCM 7050; (**D**)—*Aliarcobacter cryaerophilus* UPa 2013/13; (**E**)—*Aliarcobacter lanthieri* LMG 28517; (**F**)—*Aliarcobacter thereius* LMG 24488. The results are presented as a mean ± standard deviation. Statistical significance between the groups was assessed by ANOVA at 5% level.

The antimicrobial activity of olive oil extracts against selected bacterial strains was observed. As shown in Figure 2, the time kill curves had a declining trend for all bacterial strains for each extract. However, the strongest antimicrobial effect was observed with extra-virgin olive oil extracts (*p* < 0.05). That can probably also be related to the highest total content of polyphenols and amount of individual analyzed compounds. A significant decrease in viable cells was observed after only 30 min of exposure. In this respect, Spanish extra-virgin olive oil was the most inhibitory, in which the complete inhibition of *Al*. *butzleri* UPa 2013/30, *Al*. *cryaerophilus* CCM 7050, and *Al*. *cryaerophilus* UPa 2013/13 strains was recorded after just 30 min of exposure. *Al*. *thereius* LMG 24488 was evaluated as the most resistant strain, and survived after exposure for 60 min, but in a very small number of living cells. A slightly lower antimicrobial effect was observed for Greek oil, with some strains exhibiting a more gradual decrease in the number of viable and culturable cells. However, complete inhibition was observed for most strains after an exposure time of 60 min, with the exception of *Al*. *lanthieri* LMG 28,517 and *Al*. *cryaerophilus* CCM 7050 strains, for which viable cell counts of 2.2 or 2.3 log CFU/mL, respectively, were recorded after 60 min of exposure. An interesting difference was observed among the strains of *Al*. *cryaerophilus*. The isolate from the wastewater sample (*Al*. *cryaerophilus* UPa 2013/13) was significantly more sensitive to the effects of the extract (after 30 min of exposure, there was an approximately three-log decrease in viable cells) compared to the collection strain (*p* < 0.05).

Extracts from other oil samples exhibited less antimicrobial efficacy than the extra-virgin olive oil extracts. For mixed sunflower oil with extra-virgin oil (Ondoliva), the most effective inhibition occurred in the *Al*. *butzleri* UPa 2013/30 strain (total inhibition after 3 h of exposure). However, after an exposure time of 30 min, the overall decrease in viable cell counts was only 0.2 log CFU/mL. Further, the other strains were only inactivated after exposure for 6–24 h. In the presence of refined pure olive oil extract, complete inhibition of most of the tested strains was not observed until after 24 h of exposure. The exception was the strain *Al*. *thereius* LMG 24488, in which there was a rapid decline in viable cells after an exposure of 1 h, and complete inhibition after 6 h of exposure (a four-log decrease). In contrast, the strain *Al*. *butzleri* UPa 2013/30 was not inactivated even after 24 h of exposure, when the number of viable cells was still recorded at 2.53 log CFU/mL.

The extract of olive pomace oil exhibited the lowest antimicrobial efficacy compared to all the other samples. The most sensitive was *Al*. *thereius* LMG 24488, which was inactivated after 6 h of exposure. However, for the other strains included in the study, a maximum decrease in viable cells of up to 1.71 log CFU/mL was observed within 6 h. The strain of *Al*. *butzleri* UPa 2013/30 was not even inactivated after 24 h of exposure, similar to the refined olive oil sample.

### 2.4. Antimicrobial Activity of Oil Extracts—Time Kill Curves of WEOO

**Figure 3:**
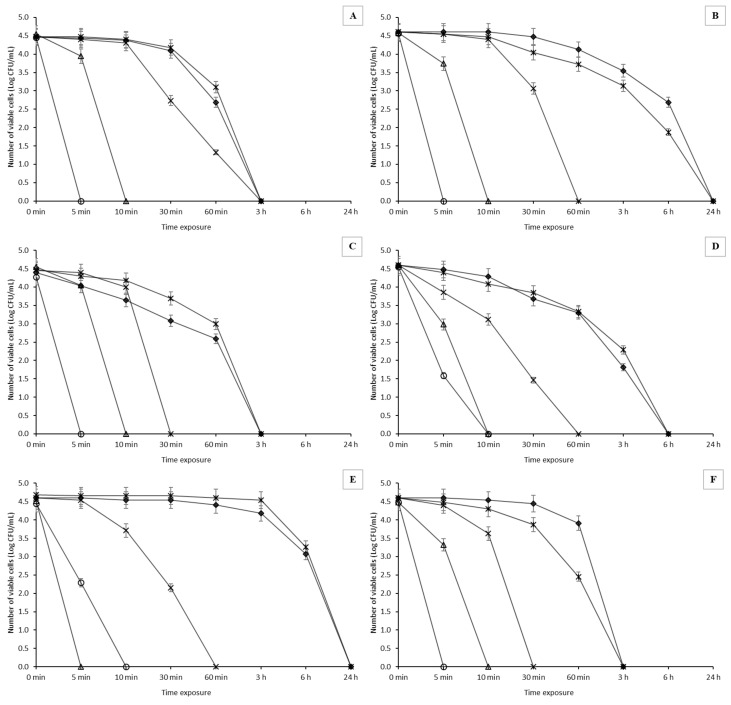
Survival of *Arcobacter*-like strains in the presence of WEOO at a sample concentration of 90%. Spanish extra-virgin olive oil (circle); Greek extra-virgin olive oil (triangle); blended sunflower and extra-virgin olive oil (times); pure olive oil (diamond); olive pomace oil (asterisk). (**A**)—*Aliarcobacter butzleri* CCUG 30484; (**B**)—*Aliarcobacter butzleri* UPa 2013/30; (**C**)—*Aliarcobacter cryaerophilus* CCM 7050; (**D**)—*Aliarcobacter cryaerophilus* UPa 2013/13; (**E**)—*Aliarcobacter lanthieri* LMG 28517; (**F**)—*Aliarcobacter thereius* LMG 24488. The results are presented as a mean ± standard deviation. Statistical significance between the groups was assessed by ANOVA at 5% level.

Spanish extra-virgin olive oil (Ballester) with the highest total polyphenol content again demonstrated the highest antimicrobial effect (*p* < 0.05). The extract from this oil inhibited most of the monitored strains after only 5 min of exposure (see Figure 3). After this very short exposure, viable cells decreased by up to 5 log CFU/mL. The only exception was the strain *Al*. *lanthieri* LMG 28517, in which a decrease in viable cells of 2 log CFU/mL was observed after 5 min of exposure, but after a total of 10 min of exposure, the cells were already completely devitalized.

None of the *Arcobacter*-like strains were able to survive after 10 min in water extract of Greek extra-virgin olive oil. With the *Al*. *lanthieri* LMG 25,817 strain, the survival was completely suppressed after only 5 min of exposure. The bland oil extract (Ondoliva) inhibited the monitored arcobacters after exposure for 30–180 min. The most resistant strain was *Al*. *butzleri* CCUG 30484, in which mild survival was still observed after 60 min of exposure, although with a four-log decrease in CFU/mL compared to the initial cell count. In the presence of refined olive oil extract, the fastest inhibition occurred in *Al*. *thereius* LMG 24,488 and *Al*. *cryaerophilus* CCM 7050 (a decrease of 4–5 log CFU/mL). However, with *Al*. *butzleri* UPa 2013/30, and especially *Al*. *lanthieri* LMG 28517, survival was observed after 6 h of exposure and complete suppression was noted after 24 h of exposure.

The lowest antimicrobial effect was observed in the olive pomace oil extract, with complete inhibition after 3–24 h of exposure. E.g., in the *Al*. *lanthieri* LMG 28,517 strain, there was almost no decrease in viable cells over 6 h of exposure.

### 2.5. Biofilm Formation Assay in the Presence of BEOO

**Figure 4:**
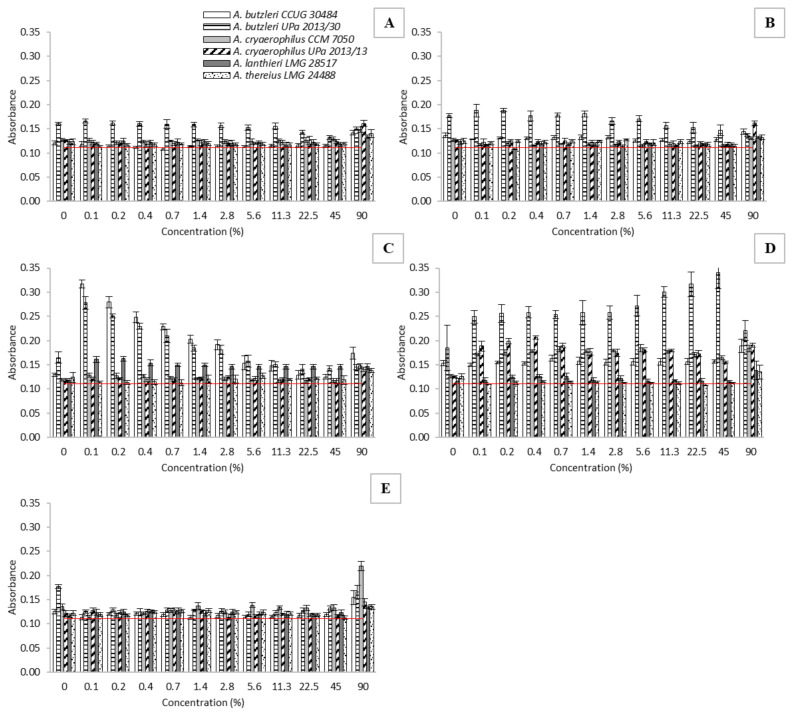
Biofilm formation in the presence of BEOO at a concentration of 90%. (**A**)—extra virgin olive oil (Ballester); (**B**)—extra virgin olive oil (Kyosos); (**C**)—blended sunflower and extra-virgin olive oil (Ondoliva); (**D**)—pure olive oil (Borges); (**E**)—olive pomace oil (Ondoliva). The horizontal red line represents the influence of BHI broth (values under horizontal line—biofilm negative; values above line—biofilm positive). The results are presented as a mean ± standard deviation. Statistical significance between the groups was assessed by ANOVA at 5% level.

The biofilm-forming ability of *Arcobacter*-like strains was monitored in the presence of various oil extract concentrations (Figure 4). According to our preliminary results, all the tested strains are able to form a biofilm. Among the extracts, a different effect on biofilm formation was observed for *Arcobacter*-like strains. In general, it can be stated that the highest tested concentration of extracts usually led to increased biofilm formation of the studied strains. This is probably due to the increased stress acting on the cells, in which the bacteria could no longer survive in planktonic form and immediately formed a biofilm structure. Thus, bacteria try to prevent these unfavorable conditions by forming a biofilm.

The biofilm formation activity of *Al*. *butzleri* and *Al*. *cryaerophilus* strains (collected and isolated strains) in the presence of individual samples was comparable. The highest biofilm formation (without the presence of extracts) was observed in the strain *Al*. *butzleri* UPa 2013/30, and this ability did not change significantly at different concentrations of extra-virgin olive oil extracts (see Figure 4A,B). Further, in the presence of a mixture of sunflower and olive oil, there was a significant increase in biofilm formation at the lowest concentration (0.1%). However, with higher concentrations of the extract, there was a significant decrease in biofilm activity. A similar trend was also observed for *Al*. *butzleri* CCUG 30484. With refined olive oil, there was an increase in biofilm formation in some strains, essentially over the entire concentration range (0.1–90%). Conversely, the strain *Al*. *butzleri* UPa 2013/30 exhibited increasing biofilm activity up to a 45% concentration of the extract of Borges oil.

An interesting course of the dependence of biofilm formation on the concentration of BEOO extract was recorded for *Al*. *thereius* LMG 24488. An inhibition of biofilm formation was observed in this strain in the presence of lower concentrations of BEOO extract from refined olive oil (0.1–45%) and blended extra-virgin olive oil and sunflower oil (0.1–0.7%). However, at higher concentrations, there was already a significant increase in biofilm formation, even to a higher level than that observed without the effect of the given extract. A strong increase in biofilm formation was observed in olive pomace oil extracts at the highest concentration (A_595_ = 0.22) in the strain *Al*. *cryaerophilus* CCM 7050. In this case, biofilm formation almost doubled compared to the initial state without the influence of the monitored extract.

### 2.6. Biofilm Formation Assay in the Presence of WEOO

**Figure 5:**
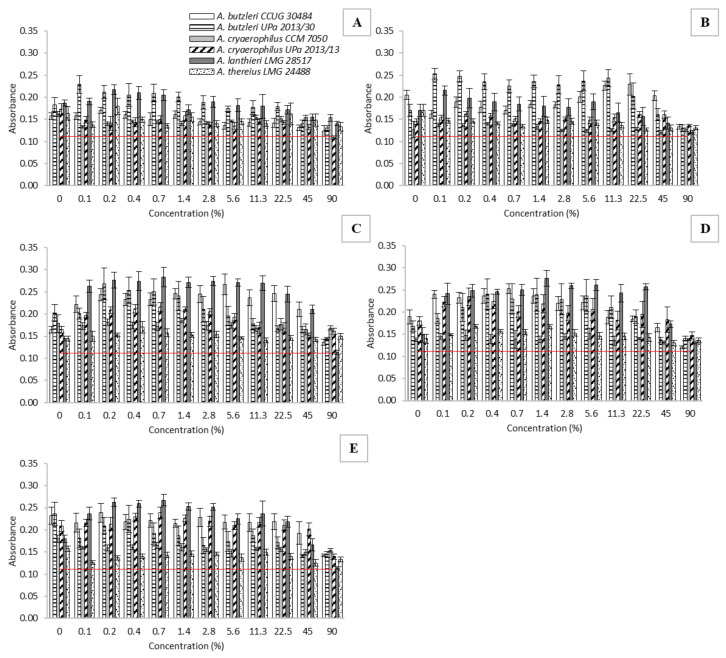
Biofilm formation in the presence of WEOO at a concentration of 90%; (**A**)—extra virgin olive oil (Ballester); (**B**)—extra virgin olive oil (Kyosos); (**C**)—blended sunflower and extra-virgin olive oil (Ondoliva); (**D**)—pure olive oil (Borges); (**E**)—olive pomace oil (Ondoliva). The horizontal red line represents the influence of BHI broth (values under horizontal line—biofilm negative; values above line—biofilm positive). The results are presented as a mean ± standard deviation. Statistical significance between the groups was assessed by ANOVA at 5% level.

The biofilm-forming ability of *Arcobacter*-like strains was monitored in the presence of various oil extract concentrations (Figure 5). According to our preliminary results, all the tested strains are able to form a biofilm at different levels. Undoubtedly, pH, which is not adjusted in any way, also plays an important role in WEOO extracts (unlike BEOO extracts in a buffer environment). The pH of WEOO extracts is in the basic range (pH 8.73) and plays an important role in the influence of extracts on biofilm formation. As the concentration of the non-buffered extract increased, there was usually an increase in biofilm activity. These results suggest that WEOO extracts contain substances that promote biofilm formation. In contrast, the highest concentrations of oil extracts led to the suppression of the ability to form a biofilm. E.g., the biofilm formation of *Al*. *butzleri* UPa 2013/30 was extremely increased in the environment of lower concentrations of extra-virgin olive oil extracts (this is especially evident for the Greek extra-virgin olive oil sample; see Figure 5B). An increased biofilm formation of up to 50% was observed in this strain compared to the biofilm formation without the influence of oil extracts. A very similar trend can be observed for the *Al*. *lanthieri* LMG 28,517 strain. In contrast, in a 90% WEOO extract concentration, there was a significant reduction in biofilm formation, which approached the very limit of biofilm positivity. A similar trend in the biofilm behavior of the monitored bacterial strains was also observed with the other tested WEOO extracts.

## 3. Discussion

The increased resistance of bacteria to antimicrobials is a matter of great concern worldwide; therefore, monitoring the antimicrobial resistance of microorganisms is especially important [24]. Previous studies confirm that many natural matrices and extracts have antimicrobial activity. Many resistant bacteria are found in the biofilm structure, which is up to a thousand times more resistant to antimicrobials than planktonic cells [4] and can colonize a variety of surfaces and persist in a variety of environments [25].

The aim of this study was to provide information on the biological activity of oil extracts on the inhibition and biofilm formation ability of *Arcobacter*-like microorganisms. In contrast with other edible vegetable oils, virgin olive oil possesses a considerable amount of phenolic compounds with a beneficial effect on human health [26,27]. Furthermore, a strong bactericidal effect of olive oils against certain foodborne pathogens has recently been reported [19,28]. The findings also confirm the possibility of using olive oils as a food preservative [19]. However, olive oils also exhibited inhibitory activity against some beneficial microorganisms. Extracts prepared from oils also exhibit certain biological effects [16,19]. The available data from the literature show that the antibacterial effect of virgin olive oils is generally higher against Gram-positive bacteria than Gram-negative bacteria [16,29]. It has been previously documented that antimicrobial activity is highest in extra-virgin olive oils, followed by other types of olive oils and olive pomace oils [19,30]. A significant inhibitory effect was also observed for *Arcobacter*-like species, especially for extra-virgin olive oil extracts, which have the highest amount of phenolic compounds. In this case, complete inhibition was observed after only 5 min of exposure in WEOO. However, the bactericidal components of olive oil can be degraded or converted during the refining process or ingestion. Significant stability of phenolic compounds in the simulated gastric juice environment was confirmed, even after more than 4 h [31]. Of course, it also depends on the amount of oil consumed [19].

In the literature, the biofilm formation ability of *Arcobacter*-like strains has been confirmed by many studies [32,33,34]. Biofilm activity differs among strains, and the environment also has a great influence on biofilm formation [35]. Our results show that some *Arcobacter*-like strains are capable of intensive biofilm formation, even in the presence of oil extracts that exhibit a bactericidal effect against planktonic cells. A significant increase in biofilm formation in *Arcobacter*-like species was observed, especially for BEOO extracts with the highest concentration (90%), although the inhibitory effect on planktonic cells was weaker than for WEOO extracts. On the contrary, at lower concentrations of WEOO extracts, a significant increase in biofilm formation was observed, with a gradual decrease at higher concentrations. In this case, these are extracts with a higher inhibitory effect on planktonic cells. It can be stated that this is the reaction of microorganisms to environmental stress conditions [34]. Since there is no single biofilm formation mechanism, different strategies are needed to improve the antimicrobial efficacy of different matrices [36]. Natural extracts have a high content of potentially antimicrobial compounds with the possibility of influencing biofilm formation [37]. The mechanisms leading to the reduction of biofilm formation through inhibition of quorum sensing are widely studied [37,38].

As the antimicrobial effects of olive oils are higher than those of some other oils, components other than the fatty acid content are believed to contribute to the antimicrobial activity. Phenolic compounds have been confirmed to be a major source of the compounds with antimicrobial potential in olive oils [19,39]. The antimicrobial activity of phenolic compounds obtained from olive oil extracts has been confirmed by a number of studies [30,40,41]. It is necessary to emphasize the large differences in composition between varieties of olive oils, such as virgin olive oil, olive oil, and olive pomace oil. According to our results, olive pomace oil contains a significantly lower content of phenolic substances than other oils (*p* < 0.05). This is in correspondence with previous studies [19,30]. The antimicrobial activity of olive oils has been attributed to oleuropein and 2-(3,4-dihydroxyphenyl)ethanol content in particular, for many years [41,42,43]. However, in recent years, the effect of other compounds, such as the dialdehydic form of decarboxymethyl oleuropein aglycon and the dialdehydic form of decarboxymethyl ligstroside aglycon, has become evident [19,44].

Medina et al. (2006) evaluated that the effect of oil extracts on the survival of selected microorganisms depends on the extraction agent and its amount. The content of phenolic compounds with antioxidant and antimicrobial activity in oil or its extracts also depends on their polarity and chemical structure [40]. Compounds that can diffuse into the aqueous medium (distilled water or buffer) are predominantly present in the prepared oil extracts. According to our results, oleuropein isomers and derivatives were better extracted into distilled water and found in higher amounts in extra-virgin olive oil extracts (52.6 mg/L). As mentioned above, the contents of tyrosol, hydroxytyrosol, and oleuropein derivatives were evaluated as the dominant compounds in the given extracts. Other studies have also confirmed the highest content of oleuropein and its derivatives in olive oils [39]. Oleuropein isomers and derivatives have previously been reported to probably include, in particular, the dialdehyde forms of oleuropein aglycone and ligstroside aglycone [45]. The total phenolic content determined by the Folin–Ciocalteu method was found to be slightly higher in the pomace oil extract compared to the refined olive oil. However, a slightly higher antimicrobial effect was observed with the refined olive oil extract.

## 4. Materials and Methods

### 4.1. Olive Oils and Sample Preparation

Olive oils of different quality and origin were purchased from local distributors in the Czech Republic. Information about the samples and their geographical origin is listed in Table 4. The olive oils were stored in the dark at room temperature, and experiments were performed immediately after opening the bottles. The extracts were prepared via extraction in phosphate-buffered saline (buffered extract; BEOO) and in distilled water (non-buffered extract; WEOO). Briefly, ten grams of olive oil were mixed with 10 mL of phosphate-buffered saline with a pH adjusted to 7, or with 10 mL distilled water at room temperature for 5 min with regular vortexing. After centrifugation at 9000 rpm for 3 min, the aqueous phase was collected for other experiments. Prior to the chromatographic analysis, the extracts were filtered through a syringe membrane nylon filter with a pore diameter of 0.45 µm (Labicom, Olomouc, Czech Republic).

### 4.2. Chemicals

The acetonitrile for chromatographic analysis (purity of HPLC grade), ammonium acetate (≥99%), formic acid (≥98%), gallic acid (≥98%), and Folin–Ciocalteu reagent were obtained from Sigma-Aldrich (St. Louis, MO, USA). Demineralized water was obtained by purifying distilled water in a Milli-Q water-purification system (Millipore, Bedford, MA, USA). Hydroxytyrosol (≥90%), tyrosol (≥95%), and oleuropein (≥98%) were purchased from Merck KGaA (Darmstadt, Germany). Sodium carbonate (p.a.) was obtained from Penta (Praha, Czech Republic).

### 4.3. Chromatographic Analysis

The HPLC system for chromatographic analysis consisted of a vacuum degasser DG 3014 (Ecom, Prague, Czech Republic), two model 582 chromatographic pumps (ESA, Chelmsford, MA, USA), and an electrochemical 8-channel CoulArray 5600A detector (ESA Chelmsford, MA, USA) was used. A Gemini C18 chromatographic column (150 mm × 3 mm I.D., 3 µm particle size) obtained from Phenomenex (Torrance, CA, USA) was used for the extracts analysis. We used an aqueous solution of ammonium acetate (5 mM) acidified with formic acid for pH~3 (mobile phase A) and acetonitrile (mobile phase B), at a flow rate of 0.4 mL/min with linear gradient elution, as follows: 0 min 0–30 min: 5–60% of mobile phase B. The sample volume was 10 µL, and a separation temperature of 40 °C was used. Working potentials of 200–900 mV (step 100 mV) were applied to the eight electrochemical cells of the detector.

### 4.4. Total Polyphenol Content (TPC) Determination

The total phenolic content of the extracts was determined using Folin–Ciocalteu assay [23]. The external calibration was carried out using different concentrations of gallic acid in the range of 0.1–50 mg/L. In brief, 1 mL of 95% ethanol, 5 mL of distilled water, and 0.5 mL of Folin–Ciocalteu reagent were added to 1 mL of extract. After 5 min, 1 mL of 5% sodium carbonate solution was added. This mixture was left for 60 min at laboratory temperature. Furthermore, the absorbance of the mixture was measured using a Genesys 50 UV-VIS spectrophotometer (ThermoFisher Scientific, Waltham, MA, USA) at a wavelength of 765 nm against the blank. The results were expressed as gallic acid equivalents (GAE). All determinations were performed four times, and the results are expressed as a mean with standard deviations.

### 4.5. Antimicrobial Effect of WEOO and BEOO

The microorganisms used in this study were as follows: *Al*. *butzleri* CCUG 30484, *Al*. *butzleri* UPa 2013/30, *Al*. *cryaerophilus* CCM 7050, *Al*. *cryaerophilus* UPa 2013/13, *Al*. *lanthieri* LMG 28517, and *Al*. *thereius* LMG 24488. Strains were obtained from the Czech Collection of Microorganisms (CCM, Brno, Czech Republic), Culture Collection University of Göteborg (CCUG, Göteborg, Sweden), Belgian Co-ordinated Collections of Microorganisms (LMG, Ghent, Belgium), or isolated at the University of Pardubice (UPa, Pardubice, Czech Republic). Cultures were grown on Tryptone Soya agar (TSA, HiMedia, Mumbai, India) for 48 h at 30 °C before testing. Cells were suspended in physiological saline to a value of 0.5 on the McFarland scale (3–9 × 10^8^ CFU/mL). The cell suspension was then diluted to an appropriate cell density before each testing.

Bacterial suspensions at a cell density of 10^6^ CFU/mL were mixed with WEOO or BEOO with the sample of oil extract at a final concentration of 90% in a test tube in a 1:9 ratio. After exposure (0; 5; 10; 30; 60 min, and 24 h) at room temperature, time kill curves were obtained by counting surviving colony-forming units on Mueller–Hinton agar (HI-MEDIA, India) after incubation at 30 °C for 24 h under aerobic conditions. In some cases, additional exposure time was necessary. The experiment was performed in duplicate, and all experiments were independently repeated 3 times.

### 4.6. Biofilm Formation Testing in Presence of WEOO and BEOO

Biofilm formation in the presence of WEOO and BEOO was monitored in flat-bottomed microtiter plates (SPL Life Sciences, Pocheon-si, Korea), as previously described [46]. Briefly, the tested concentrations in the range of 0–90% were prepared in brain hearth infusion (BHI, Himedia, India). A bacterial culture was added to the individual positions of the microtiter plate to obtain a final cell density of 1.5 × 10^8^ CFU/mL. After cultivation at 30 °C for 24 h under aerobic conditions, the microtiter plate was washed with sterile distilled water and dried. Biofilm fixation was performed with 2% sodium acetate (15 min). Attached cells (biofilm structure) were stained with 100 µL of filtered 1% crystal violet solution (Sigma-Aldrich, St. Louis, MO, USA) that was incubated for 15 min at room temperature. Subsequently, the unbound crystal violet was washed out carefully with sterile distilled water. Thereafter, the biofilm-associated crystal violet was solubilized with 96% ethanol. Then, 100 µL was taken from each well and the absorbance was measured in a new plate at 595 nm (Infinite M200, Tecan, Männedorf, Switzerland). The biofilm formation in the presence of BHI is represented by red lines. There were 8 wells in each experiment, and the experiments were independently repeated 3 times. The level of biofilm formation of the *Arcobacter*-like strains was categorized, according to a previously described classification system [24], as non-adherent (OD ≤ OD_C_) or biofilm-forming strains (OD > OD_C_), where OD_C_ (*cut-off* OD) is defined as three standard deviations above the mean OD of the negative control (blank value). The measured and calculated OD/OD_C_ (0.111/0.120) values were the same for all measurements.

### 4.7. Statistical Analysis

The obtained values were statistically evaluated using Excel 2016 (Microsoft, Redmond, WA, USA) and Statistica 12 (StatSoft, Tulsa, OK, USA). Extreme values were tested with the Dean–Dixon Q test, and all remoteness values were excluded with 95% probability. Median and standard deviation were calculated from the remaining values. A possible source of error, which resulted from insufficient dye washing in the biofilm staining leading to increased absorbance, was also considered. The significance of results was evaluated by one-way analysis of variance (ANOVA) with a significance level of *p* < 0.05.

## 5. Conclusions

Increasing bacterial resistance to known antibiotics is a growing problem. Bacteria that are capable of forming a biofilm are even more resistant to inactivation. Due to this fact, it is necessary to explore new substances that support the elimination of biofilm-forming microorganisms. Plant extracts and their extracts are often associated with an inhibitory effect on biofilm-forming bacteria. To our knowledge, this is the first in vitro study to evaluate the biological effects of olive oil extracts on *Arcobacter*-like species. The results obtained in this study suggest that olive oil extracts are able to inhibit the growth and biofilm formation of *Arcobacter*-like strains. The cells were very effectively inactivated after a short exposure of the cells in the environment of oil extracts. The antimicrobial effect of the extracts corresponds to the content of phenolic compounds determined by HPLC-CoulArray. Biofilm formation is a highly undesirable property of microorganisms from an industrial and healthcare perspective. A significant effect of the extracts on biofilm formation was observed. An increase in biofilm formation was observed at lower concentrations of extracts; however, at higher concentrations, there was usually a decrease in biofilm formation. On the basis of the described results, it is clear that it would be interesting to further deepen knowledge in this area with the aim of possible use of olive oil extracts as antimicrobial substances.

## Figures and Tables

**Table 1:** Chromatographic characteristics of standards.

Compound	LOD (mg/L)	LOQ (mg/L)	Calibration Range (mg/L)	Regression Equation	R^2^
Hydroxytyrosol	0.068	0.227	0.5–25.0	y = 16.796x − 5.9317	0.9714
Tyrosol	0.025	0.083	0.5–25.0	y = 17.622x + 18.891	0.9654
Oleuropein	0.126	0.423	0.5–25.0	y = 3.833x + 0.1662	0.9963

LOD—limit of detection; LOQ—limit of quantification; R^2^—correlation coefficient.

**Table 2:** Contents of followed compounds (mg/L) in WEOO and BEOO.

	Chemical Compound	Extra-Virgin Olive Oil (Ballester)	Extra-Virgin Olive Oil (Kyosos)	Blended Olive and Sunflower Oil (Ondoliva)	Refined Olive Oil (Borges)	Olive Pomace Oil (Ondoliva)
WEOO	Hydroxytyrosol	9.82 ± 0.12 ^a^	9.01 ± 0.24 ^b^	0.40 ± 0.01 ^c^	<LOD	<LOD
	Tyrosol	8.93 ± 0.12 ^a^	10.17 ± 0.06 ^b^	<LOQ	<LOD	<LOD
	Oleuropein derivates	52.64 ± 1.84 ^a^	29.20 ± 1.95 ^b^	0.99 ± 0.15 ^c^	<LOD	<LOD
BEOO	Hydroxytyrosol	9.97 ± 1.81 ^a^	7.97 ± 0.08 ^b^	0.93 ± 0.08 ^c^	<LOD	<LOD
	Tyrosol	12.30 ± 2.04 ^a^	12.49 ± 0.72 ^a^	2.70 ± 0.07 ^b^	<LOD	<LOD
	Oleuropein derivates	10.26 ± 0.44 ^a^	7.69 ± 0.01 ^b^	0.79 ± 0.15 ^c^	<LOD	<LOD

The results are presented as a mean ± standard deviation. Statistical significance between the groups was assessed by ANOVA at 5% level. Means in the same row with different superscript small letters differ significantly (*p* < 0.05). LOD—limit of detection; LOQ—limit of quantification.

**Table 3:** Total polyphenol content (GAE mg/L) in WEOO and BEOO.

	Extra-Virgin Olive Oil (Ballester)	Extra-Virgin Olive Oil (Kyosos)	Blended Olive and Sunflower Oil (Ondoliva)	Refined Olive Oil (Borges)	Olive Pomace Oil (Ondoliva)
WEOO	79.82 ± 5.04 ^a^	43.50 ± 2.92 ^b^	11.09 ± 0.80 ^c^	0.36 ± 0.03 ^d^	0.49 ± 0.02 ^e^
BEOO	93.23 ± 2.84 ^a^	49.05 ± 1.65 ^b^	15.22 ± 0.80 ^c^	0.43 ± 0.01 ^d^	0.47 ± 0.01 ^d^

The results are presented as a mean ± standard deviation. Statistical significance between the groups was assessed by ANOVA at 5% level. Means in the same row with different superscript small letters differ significantly (*p* < 0.05).

**Table 4:** List of oil samples.

Grade of Olive Oil	Trademark	Country of Origin	Manufacturer/Distributor
Extra-virgin olive oil	Ballester	Spain	Juan Ballester Rosés Sucesores SA, Tortosa, Spain
Extra-virgin olive oil	Kyosos	Greece	HM Weihs, Vienna, Austria
Blended sunflower and extra-virgin olive oil	Ondoliva	Spain	Urzante SL, Tudela, Spain
Refined olive oil and extra-virgin olive oil	Borges	Spain	Borges Branded Foods SLU, Reus, Spain
Olive pomace oil	Ondoliva	Spain	Urzante SL, Tudela, Spain

## Data Availability

Not applicable.

## Sample Availability

Samples of the oil extracts are available from the authors.
